# Grip and load force coordination in cyclical isometric manipulation task is not affected by the feedback type

**DOI:** 10.1186/1743-0003-10-34

**Published:** 2013-04-04

**Authors:** Sabrina Tiago Pedão, José Angelo Barela, Kauê Carvalho de Almeida Lima, Paulo Barbosa de Freitas

**Affiliations:** 1 Institute of Physical Activity and Sport Sciences and Graduate Program in Human Movement Sciences, Cruzeiro do Sul University, Rua Galvão Bueno 868, 13° andar, Bloco B, São Paulo 01506-000, SP, Brazil; 2 Departament of Physical Education, Bioscience Institute, São Paulo State University at Rio Claro, Av. 24-A, 1515, Bela Vista, Rio Claro, São Paulo, SP 13506-900, Brazil

**Keywords:** Visual, Auditory, Hand function, Motor control

## Abstract

**Background:**

The relationship between normal and tangential force components (grip force – GF and load force – LF, respectively) acting on the digits-object interface during object manipulation reveals neural mechanisms involved in movement control. Here, we examined whether the feedback type provided to the participants during exertion of LF would influence GF-LF coordination and task performance.

**Methods:**

Sixteen young (24.7 ±3.8 years-old) volunteers isometrically exerted continuously sinusoidal F_Z_ (vertical component of LF) by pulling a fixed instrumented handle up and relaxing under two feedback conditions: targeting and tracking. In targeting condition, F_Z_ exertion range was determined by horizontal lines representing the upper (10 N) and lower (1 N) targets, with frequency (0.77 or 1.53 Hz) dictated by a metronome. In tracking condition, a sinusoidal template set at similar frequencies and range was presented and should be superposed by the participants’ exerted F_Z_. Task performance was assessed by absolute errors at peaks (AE_Peak_) and valleys (AE_Valley_) and GF-LF coordination by GF-LF ratios, maximum cross-correlation coefficients (r_max_), and time lags.

**Results:**

The results revealed no effect of feedback and no feedback by frequency interaction on any variable. AE_Peak_ and GF-LF ratio were higher and r_max_ lower at 1.53 Hz than at 0.77 Hz.

**Conclusion:**

These findings indicate that the type of feedback does not influence task performance and GF-LF coordination. Therefore, we recommend the use of tracking tasks when assessing GF-LF coordination during isometric LF exertion in externally fixed instrumented handles because they are easier to understand and provide additional indices (e.g., RMSE) of voluntary force control.

## Background

The ability to use one or both hands to grasp and manipulate objects is essential for performing innumerous daily living activities and, consequently, is important for maintaining an independent lifestyle. Therefore, several studies have explored different aspects of hand function. Specifically, a largely used and elegant experimental paradigm has been applied to investigate the relationship between force components acting on the digits and object surface interface during object manipulation. The force component acting tangentially on the digits-object interface, referred to as load force (LF), tends to cause slippage of the handheld object, which is prevented by the exertion of force perpendicularly to the object surface, which has been termed grip force (GF) [1-3].

The close relationship established between GF and LF during object manipulation is a striking evidence of the central nervous system’s (CNS) ability to predict the effects of individuals’ own actions [2,4-6]. This coupling has been widely investigated during manipulation tasks that involve lifting a grasped object [1], moving a handheld object upward and downward discretely [7] or continuously [5,8], and isometrically applying sinusoidal LF profiles on an externally fixed object [4,6,9]. This highly coupled relationship is characterized by a parallel change of GF and LF with virtually no time delay. During tasks in which LF changes (e.g., lifting, transporting, and shaking a handheld object) this parallel change of GF and LF ensure an economical exertion of GF [1,4,5,7,9].

Investigations of GF-LF coupling using continuous changes in LF during manipulation of free moving and fixed objects have provided consistent support for the use of predictive strategies by CNS in healthy [4,5] and neurological individuals [10-12]. However, the use of an externally fixed object could be advantageous when compared with the use of a free moving one because researchers could easily regulate LF magnitude and frequency. Also, additional information about the CNS current condition and about the adopted control strategies related to visuomotor coordination could be obtained by assessing the individual’s ability to control LF (i.e., task performance) over the task time course. Hence, a common methodological feature of this type of experiment is to provide both the real time visual feedback of the exerted LF and any type of information regarding the prescribed LF magnitude and frequency to guide participants in the task execution. For example, in two studies performed in fixed (or quasi-fixed) objects, visual information about the prescribed LF magnitude and frequency and about the currently exerted LF was presented in oscilloscopes and computer screens [4,10,13]. Moreover, in other studies [6,9], visual information about the prescribed LF magnitude was shown in a computer screen (i.e., visual feedback) while information about LF frequency was given by a metronome (auditory feedback).

To our knowledge, no one compared the effects of exerting cyclical isometric LF using different types of feedback on task performance as well as on GF scaling and GF-LF coupling. The comparison between two different types of feedback (visual vs. visual plus auditory) is a relevant methodological aspect when designing studies aiming to investigate the relationship between GF and LF and to assess hand function in healthy and hand-impaired individuals. Regarding task performance, we could expect differences between feedback conditions because any motor task that requires simultaneous processing of visual and auditory feedback could be considered more complex than a task that requires processing of a single source of feedback because individuals need to deal with two different sources of information, which increases, for example, attentional demands. Thus, looking from this perspective, individuals with attention deficits (e.g., old adults, Parkinson and cerebellar patients, and specific group of children) would benefit from a simplified experimental approach as the proposed by this study. Conversely, regarding GF scaling and GF-LF coupling, the need for accuracy throughout the whole task imposed by a visual task could be a burden on the CNS and it would make the system to change the adopted control strategy, that is, increase GF magnitude and reduce GF-LF coupling.

Thus, before recommending one experimental approach in detriment of another we need to investigate whether they produce different outcomes in variables related to GF-LF coordination as well as task performance. Therefore, we examine in this study whether the type of feedback received by the individuals during exertion of oscillatory isometric LF profile would influence task performance and GF scaling, as well as GF-LF coordination. If we find differences in task performance and, mainly, in GF-LF coordination we would have to take into consideration this fact before selecting one type of feedback that interfere less with GF-LF coordination. Alternatively, if we find no differences in those variable we could claim that the type of feedback received by the participants is not influential in GF-LF coordination and could recommend the use of either one based upon the advantages that one type of feedback has comparing to the other.

## Methods

### Participants

Sixteen healthy and right-handed individuals (eight males), ranging from 19 to 33 years of age (mean ± S.D.: 24.7 ±3.8 years-old) participated in the study. Their participation was conditioned to the signature of an informed consent form. All experimental procedures and the informed consent form given to and signed by the participants were approved by the research ethics committee of the Cruzeiro do Sul University. This study was conducted in accordance with the Declaration of Helsinki.

### Instrumentation

**Figure 1:**
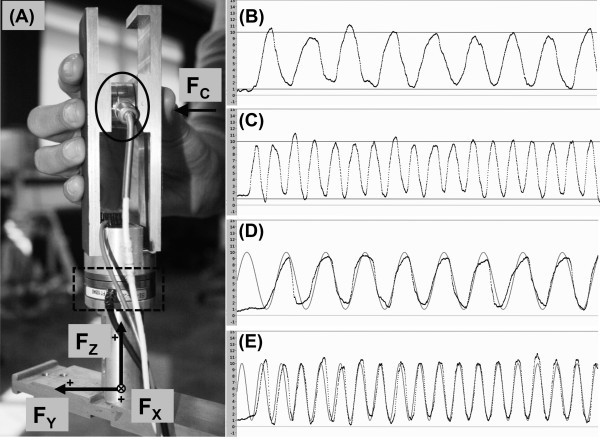
**Instrumented handle photography and representation of all four experimental conditions.** (**A**) Photography of the handle used in the experiment and representation of the force components recorded by the single-axis (ellipse) and multi-axis F/T transducers (dashed rectangle). (**B-E**) The vertical force component of LF (F_Z_) time-series (black lines) of trials performed at 0.77 Hz (**B** and **D**) and at 1.53 Hz (**C** and **E**) in the targeting condition (**B** and **C**), in which two horizontal lines (gray lines) displayed in the computer monitor represented the prescribed upper and lower targets, and in the tracking condition (**D** and **E**), in which a sinusoidal template (gray line) shown in the computer monitor represented the prescribed target.

An instrumented handle consisting of two parallel aluminum plates (15x4 cm) connected with each other by a single-axis force transducer (LPM-530, Cooper Instruments and Systems, USA) and two aluminum pieces with a multi-axis force and torque (F/T) transducer (Mini40, ATI, USA) in between them (forming the base of the handle) was used in the study (Figure 1A). The opposing handle grasping surfaces were 5 cm apart and were covered with sandpaper (320 grit). The single-axis force transducer recorded the compression force (F_C_) exerted by the tip of the thumb against the handle, while the F/T transducer recorded all three force and torque components applied against the handle by the digits. The horizontal force components acting perpendicularly to the handle contact areas (i.e., F_C_ recorded by the single-axis and F_Y_ recorded by the multi-axis F/T transducer) were used to calculate the grip force [GF = (F_C_ + (||F_C_-F_Y_||))/2], i.e., the average force exerted against the two sides of the handle [9,14]. The vertical (F_Z_) and horizontal (F_X_) force components recorded by the F/T transducer, both tangential to the handle surface, were used to calculate load force [LF = √(F_Z_^2^ + F_X_^2^)].

### Experimental procedure

Before starting the experiment, all participants cleaned the tip of their digits with alcohol swabs. Then, they stood upright, kept their upper arm vertically and forearm horizontally oriented, and were asked to grasp the fixed handle using the tip of their digits as shown in Figure 1A. The vertically oriented handle was rotated 45° with respect to the participants’ frontal plane to assure a comfortable wrist position. Next, the participants were asked to exert a sinusoidal pattern of the vertical component of LF (F_Z_), by isometrically pulling the handle up and relaxing up to a certain point. They were requested to do it within a prescribed range (i.e., 9 N, ranging from 1 to 10 N) at two different frequencies [46 bpm (≈0.77 Hz) and 92 bpm (≈1.53 Hz)].

The participants performed the task receiving two distinct types of feedback: *targeting and tracking*. In the *targeting* condition, they received information of the prescribed F_Z_ magnitude by two horizontal lines presented in a 19-in. widescreen computer monitor placed in front of them (Figure 1B-C) and information of the prescribed frequency by a metronome set at the above mentioned frequencies. They also received information about the current real-time F_Z_ exerted by them shown as a continuous left to right running black line. The participants were instructed to continuously exert sinusoidal F_Z_ reaching the upper and lower targets (red lines) as accurate as possible following the rhythm dictated by the metronome. In the *tracking* condition, sinusoidal templates set at similar frequencies (either 0.77 Hz or 1.53 Hz) and range (9 N, from 1 to 10 N) were individually shown in the screen (Figure 1D-E). In this condition, the participants were asked to exert oscillatory F_Z_ in order to match (i.e., superpose) the sinusoidal template, also as accurate as possible. The force range was selected based upon the fact that healthy young individuals, in the most of the daily situations in which they manipulate objects with a precision grip, do not generate high levels of tangential force due to the limitation of GF muscles to generate high magnitudes of grip to prevent slippage. Regarding the chosen frequencies, we selected a slow and a fast rate of F_Z_ change, which allowed for a “within cycle” and a “next cycle” F_Z_ corrections, respectively. Moreover, we chose 46 bpm (≈0.77 Hz) and 92 bpm (≈1.53 Hz) because the commercially available metronomes have a multiple of two divisions starting at 40 bpm.

A period of familiarization with the apparatus, magnitude, and frequency was given to the participants. Namely, the participants performed between 2 and 5 trials to be able to perform the task properly, reaching the upper and lower targets and/or tracking accurately the sinusoidal profile. After, force signals from three trials for each condition of feedback and frequency were recorded at 200 Hz and stored for further analysis. Individual trials lasted 12 s and were performed in a balanced sequence, with half of the participants starting with the targeting condition and half starting with the tracking one. The same was done for frequency. Note that participants were only instructed to exert F_Z_ by isometrically pulling up the handle and GF was not mentioned whatsoever.

### Data processing and analyses

Two customized LabView (Version 2010, National Instruments, Austin, TX, USA) routines were used for data acquisition and processing. The raw force data were low-pass filtered at 20 Hz with a fourth-order (zero-phase lag) Butterworth filter. Data from the first 4 s (phase for F_Z_ frequency and magnitude adjusts) and the last 2 s (end of trial) of each trial were not considered and removed after filtering processing. Therefore, only the data between the 4th and 10th s were analyzed. Next, dependent variables related to task performance, GF scaling, and GF-LF coupling were calculated. Task performance was assessed by absolute errors (AE) [15] of F_Z_ peaks and valleys with respect to the upper (AE_Peak_) and lower (AE_Valley_) targets, respectively. GF-LF coordination was evaluated by measures of GF scaling and GF-LF coupling. GF scaling, which represents the CNS ability to scale GF with respect to LF, was assessed by GF-LF ratio (GF/LF), calculated as the averaged GF divided by the averaged absolute LF. Also, the maximum cross-correlation coefficient (r_max_) between GF and LF and its respective time lag (positive values indicating that GF lags LF) were computed from a linear cross-correlation analysis to assess, respectively, the directional and temporal coupling between LF and GF.

### Statistical analyses

Five two-way, repeated measures, analyses of variance (ANOVA) were employed to test the effects of feedback (targeting vs. tracking) and frequency of F_Z_ exertion (0.77 vs. 1.53 Hz) and the interaction between these factors on AE_Peak_, AE_Valley_, (GF/LF), Fisher’s z transformed of the r_max_, and time lag. The significance level was set at .05.

## Results

Overall, the results showed that the participants were able to exert sinusoidal F_Z_ profiles reaching the lower and upper targets accurately (AEs in average less than 1 N) at the requested frequencies independently of the type of feedback presented. Also, very high r_max_ values (>.87) with most of time lags ranging within ± 20 ms interval were observed regardless of feedback type and F_Z_ frequency. The averaged values of the dependent variables related to task performance (AE_Peak_ and AE_Valley_), GF scaling (GF/LF) and directional and temporal GF-LF coupling (r_max_ and time lag) are presented in Table 1.

**Table 1:** **Values representing task performance (AE**_**Peak **_**and AE**_**Valley**_**), GF scaling (GF/LF), and GF-LF coupling (r**_**max **_**and time lag) in targeting and tracking feedback conditions performed at 0.77 Hz and 1.53 Hz**

		**AE**_**Peak **_**(N)**	**AE**_**Valley **_**(N)**	**GF/LF**	**Time lag (ms)**	**r**_**max**_
**Targeting**	**0.77 Hz**	0.653^b^	0.596	0.779 ^b^	4.89	0.974 ^a^
		(0.077)	(0.074)	(0.055)	(3.25)	
	**1.53 Hz**	0.753^a^	0.552	0.882 ^a^	9.27	0.962 ^b^
		(0.051)	(0.056)	(0.046)	(4.42)	
**Tracking**	**0.77 Hz**	0.573^b^	0.491	0.738^b^	6.04	0.98 ^a^
		(0.041)	(0.058)	(0.053)	(3.17)	
	**1.53 Hz**	0.733^a^	0.461	0.95^a^	9.22	0.947 ^b^
		(0.052)	(0.056)	(0.06)	(3.46)	

Note: Means and respective standard errors are shown for the first four variables and median values are shown for r_max_. Also, "a" significantly greater than "b".

### Task performance

Regarding the effect of feedback and frequency on AE_Peak_, ANOVA revealed no main effect of feedback [F(1,15) = 1.5, p > .05, η^2^ = .09] and no feedback by frequency interaction [F(1,15) = 0.15, p > .05, η^2^ = .03], but revealed that AE_Peak_ was larger at 1.53 Hz than at 0.77 Hz [F(1,15) = 10.68, p < .01, η^2^ = .42]. For AE_Valley_, however, ANOVA revealed neither main effect of feedback [F(1,15) = 2.5, p > .05, η^2^ = .14], nor of frequency [F(1,15) = 0.56, p > .05, η^2^ = .04], and no feedback by frequency interaction [F(1,15) = 0.04, p > .05, η^2^ = .01].

### GF scaling

ANOVA for GF/LF revealed neither effect of feedback [F(1,15) = 0.15, p > .05, η^2^ = .01] nor feedback by frequency interaction [F(1,15) = 2.83, p > .05, η^2^ = .16], but revealed that GF-LF ratio was higher at 1.53 Hz than at 0.77 Hz [F(1,15) = 18.04, p < .005, η^2^ = .55].

### Directional and temporal GF-LF coupling

ANOVA for r_max_ Fisher’s z transformed values revealed no main effect of feedback [F(1,15) = 0.4, p > .05, η^2^ = .03] and no feedback by frequency interaction [F(1,15) = 0.005, p > .05, η^2^ = .01], but revealed that r_max_ values were the highest at the lower frequency (i.e., 0.77 Hz) [F(1,15) = 14.3, p < .005, η^2^ = .49]. Regarding the time lag, ANOVA revealed no main effect of feedback [F(1,15) = 0.71, p > .05, η^2^ = .05] and frequency [F(1,15) = 1.04, p > .05, η^2^ = .09], and no feedback by frequency interaction [F(1,15) = 0.86, p > .05, η^2^ = .05].

## Discussion

The aim of the study was to examine whether the type of feedback received by the participants while exerting oscillatory isometric LF profile would influence task performance and GF-LF coordination. The results showed that the type of feedback received did not impact any of the variables and that it is not frequency dependent. Moreover, the results revealed the already known effect of frequency on GF-LF coordination and task performance (lower r_max_ and higher GF-LF ratio, and higher AE_Peak_ at the higher frequencies) [6,14].

Based upon these findings, someone would have no restrictions when selecting any of the feedback types tested in this study. However, the use of a tracking task could be advantageous when compared to the targeting one. Firstly, while in the targeting condition individuals need to deal with two sources of information (visual and auditory), in the tracking condition information about the prescribed LF magnitude and about LF frequency is presented simultaneously as a single entity (i.e., a sinusoidal template). Therefore, tracking would make the task easier because attention resources would be focused on a single source and not diverged in two as in a targeting task. Moreover, the tracking task would provide better guidance for task performance because, intuitively, it would be easier for children, older adults, and persons with mild neurological and orthopedic impairment to follow the instructions of tracking (i.e., superposing) a sinusoidal template than to reach the upper and lower targets during exerting LF in a sinusoidal manner following the beats of a metronome. Likewise, individuals who undergo hearing problems (e.g., old adults) could also be able to be tested when performing a tracking task. Secondly, a tracking task could provide additional and more complete estimation of task performance (i.e., force control) than a targeting one. Dependent variables such as absolute, variable and constant errors [15], related to LF peaks and valleys, could be calculated from both tasks. However, the ability to control force throughout the task execution could only be assessed in the tracking task by using, for instance, the root mean square error (RMSE) as a dependent variable.

Alternatively, someone could suggest the use of simple vertically moving horizontal bar that would oscillate upward and downward as the source of feedback about the prescribed frequency and amplitude [10] or the use of a task that involve pursuit of a sinusoidal moving target [16]. However, while those two feedback strategies could be as effective as the tracking task to provide visual guidance, they do not provide information about the continuing force path what could be useful for force magnitude and frequency corrections during task execution [17]. Also, information about force magnitude provided by a relatively rapid moving bar does not allow individual to give priority to a high level of accuracy.

There are limitations in this study that deserve to be mentioned and could be addressed in future investigations. For instance, participants exerted sinusoidal LF profile in only one force range (1–10 N). Despite Uygur and colleagues [14] having found no effect of LF magnitude in GF-LF coordination in LF ranging from 6 to 15 N during a task identical to the performed in targeting condition, the tracking task was not tested in different load conditions and the GF-LF coordination and task performance could be affected in higher and even lower LF amplitudes. In addition, we tested the participants in only two frequencies (0.77 and 1.53 Hz). During isometric LF exertion in targeting condition, Jaric and colleagues [6] found that GF-LF coupling and GF scaling is affected in relatively very high frequency (above 3 Hz). In frequencies lower than 2.5 Hz, the indices of GF-LF coordination are kept relatively stable. However, those findings could not be applied to tracking conditions. Despite we observed no difference between feedback condition at 0.77 Hz and 1.53 Hz in GF scaling and GF-LF coupling, we could not affirm that feedback condition would not affect those parameters in frequencies higher than 1.53 Hz. Nevertheless, the amplitude and frequencies selected by this study are within the range utilized by virtually all the studies that used isometric LF exertion to investigate GF-LF coordination in individuals with neurological deficits e.g., [10-12].

## Conclusion

In conclusion, due to the fact that our findings did not indicate any effect of feedback type on GF scaling and GF-LF coupling as well as on the ability to reach the prescribed upper and lower targets, we could claim that the type of feedback presented (i.e., visual and visual plus auditory) does not affect the coordination between GF and LF. However, because tracking tasks are easier to understand and provides additional indices of voluntary force control (e.g., RMSE), we recommend from now on the use of tracking tasks such the one performed in this study during experiments involving isometric LF exertion in externally fixed instrumented objects.

## Abbreviations

GF: Grip force; LF: Load force; FZ: Vertical component of LF; CNS: Central nervous system; bpm: Beats per minute; F/T: Force and torque; AE: Absolute error; GF/LF: Grip force to load force ratio; rmax: Maximum correlation coefficient obtained from the cross-correlation function; ANOVA: Analysis of variance; RMSE: Root mean square error.

## Competing interests

The authors declare that they have no competing interests.

## Authors’ contributions

STP and KCAL were responsible for data collection, processing, and analysis as well as data interpretation and drafting the article. JAB was responsible for drafting the article and revising it critically for scientific method and content. PBF was responsible for conception and design of the study. Also, he was responsible for programing the LabView routines for data acquisition and processing, for data interpretation, and for drafting the article. All authors read and approved the final version of the manuscript.
